# Optimization of building integrated energy scheduling using an improved genetic whale algorithm

**DOI:** 10.1038/s41598-024-52995-4

**Published:** 2024-01-29

**Authors:** Liming Wei, Guoqing An

**Affiliations:** College of Electrical and Computer Technology, Jilin Jianzhu University, Changchun, Jilin, China

**Keywords:** Engineering, Energy science and technology, Energy storage, Renewable energy

## Abstract

Renewable energy generation has become the general trend with increasing environmental problems. However, the instability of renewable energy generation and the diversification of user demand are highlighted and the optimization of energy scheduling has become the key to solve the problem. This study introduces an energy scheduling optimization model tailored for building integrated energy systems, encompassing elements like gas turbines, wind and solar modules, ground source heat pumps, electric vehicles, central air-conditioning, and energy storage. The model prioritizes economic efficiency and minimal carbon emissions by first collecting and pre-processing the data for the regional building conformance, and then utilizing an enhanced multi-objective genetic whale algorithm. Evaluations on a regional complex building highlighted the algorithm’s robust convergence and stability. The resulting optimized scheduling effectively balances economic and environmental concerns, reducing costs by about 92.896 yuan per day on average and reducing carbon emissions by about 0.091 tons, promoting efficient system operation, reducing costs and mitigating environmental impacts.

## Introduction

With the accelerated progress of the economy, the excessive utilization of conventional fossil fuel resources has precipitated predicaments of environmental contamination and resource scarcity. Consequently, the advancement of novel energy sources and the enhancement of the efficiency and utilization rate of renewable energy have become immediate priorities. To address this issue, the establishment of a multi-energy complementary system involving wind, solar, electricity, and heat generation is paramount. By satisfying the electricity consumption demands of users, as well as the cooling and heating requirements of buildings, the achievement of “zero scenery waste” holds profound significance in the pursuit of the vision of attaining a carbon peak by 2030 and carbon neutrality by 2060. However, the present integrated energy dispatching system encountered various deficiencies, encompassing insufficient scenery utilization, sluggish energy dispatching speed, and an inequitable energy distribution ratio. To mitigate these limitations inherent to the traditional integrated energy system, this research proposes a novel integrated building energy optimization scheduling system that leverages an enhanced multi-objective genetic whale algorithm, thereby substantially ameliorating these issues ^1,2^.

Foreign scholars have made a lot of research on the construction of integrated energy optimization scheduling for various scenarios and objectives. To address the comprehensive energy optimization and dispatching in a park setting, an enhanced dynamic programming algorithm was proposed to establish a multi-objective model. Additionally, the Stackelberg master-player game was employed to account for the interplay between the supply and demand sides, aiming to minimize the anticipated operation cost of the Park Integrated Energy System (PIES) ^3–5^. For the integrated energy dispatching optimization in isolated island environment, a day-ahead model was developed that encompasses stepped carbon trading and the integration of photothermal energy storage with hydrogen production via wind power. This model not only achieves maximum net income for conventional islands but also ensures the optimization of overall energy usage ^6,7^. Furthermore, to address the issue of low efficiency in renewable energy consumption and multi-energy complementarity, a cooperative optimization operation method known as “source-network-charge-storage” was proposed for integrated energy systems. This method takes into account both wind power consumption and operational economic benefits ^8,9^. In addition, in order to improve the energy utilization rate, more efforts are made to study the energy storage part, and a cloud energy storage model is proposed for frequency modulation and peak adjustment of the power system. The optimal energy management of the home microgrid system integrating photovoltaic and battery energy storage is proposed, and energy consumption and PV generation are managed through the integration of batteries. Both methods effectively ensure energy efficiency and balance the cost to a certain extent^10,11^. Lastly, considering the different needs of different users, the combined supply of cold, heat and electricity is introduced as the core power supply unit. An optimal scheduling approach is proposed that possesses flexibility and accounts for uncertainties in building heat load, while simultaneously considering the balancing of cold, heat, and electricity, as well as equipment constraints ^12–14^. By integrating various algorithms, the optimization of comprehensive energy scheduling for buildings is achieved. Algorithms such as the Grey Wolf algorithm, multi-objective whale algorithm, and particle swarm algorithm, among others, have demonstrated the potential to enhance energy scheduling efficiency ^15–19^. Aiming at the uncertainty of renewable energy, robust optimization is proposed, rolling optimization theory is applied to emergency energy scheduling, and weight factors are introduced into the optimization model to balance the importance of reducing and retaining power^20,21^. For the study of energy efficient building management systems, through data-driven optimization models and heating and cooling air conditioning systems, combined with indoor comfort environment, to maximize the comfort index while minimizing energy consumption^22^. In summary, the majority of the aforementioned literature focuses on comprehensive energy systems within industrial parks and isolated islands, aiming to facilitate the interconnection and conversion of electricity, heat, and gas sources. Additionally, these studies incorporate carbon trading mechanisms to account for the environmental impact of carbon emissions. On one hand, due to the inadequate energy structure prevalent in many buildings, the demand for diverse energy sources is increasing. On the other hand, the use of various equipment in buildings exhibits considerable potential to improve energy efficiency and curb carbon emissions.

Therefore, a new multi-objective optimal scheduling model is proposed in this paper, which combines genetic algorithm with improved whale algorithm and introduces fitness function to simulate and analyze building energy scheduling. Firstly, the composition of the system's equipment is analyzed, and the energy flow relationships within the system are delineated. Expanding upon the conventional combined cooling, heating, and power supply system, additional components such as wind and solar power generation systems, ground source heat pump systems, and energy storage systems are incorporated. Simultaneously, carbon emissions are introduced as one of the objective functions within the model to constrain economic costs. Finally, by analyzing and processing the collected data, the Pareto optimal solution set is obtained by using the improved multi-objective genetic whale optimization algorithm, and the optimal solution set is derived from it.

This paper is divided into four main parts, and discusses the superiority of the algorithm combined with mathematical model in solving the optimization of building energy scheduling. The first part introduces the establishment of mathematical models of each part of the system and the setting of objective function constraints. The next one introduces the optimization algorithm and the improvement and innovation of the algorithm. The third part analyzes the energy scheduling of the same set of data collected under different algorithms and obtains the load running diagram. The fourth part reviews the optimization objectives and discusses the adaptability of the algorithm in building energy optimization scheduling. Finally, the application prospect of artificial intelligence algorithm in building integrated energy optimization scheduling is prospected.

## Building integrated energy optimization scheduling model

**Figure 1:**
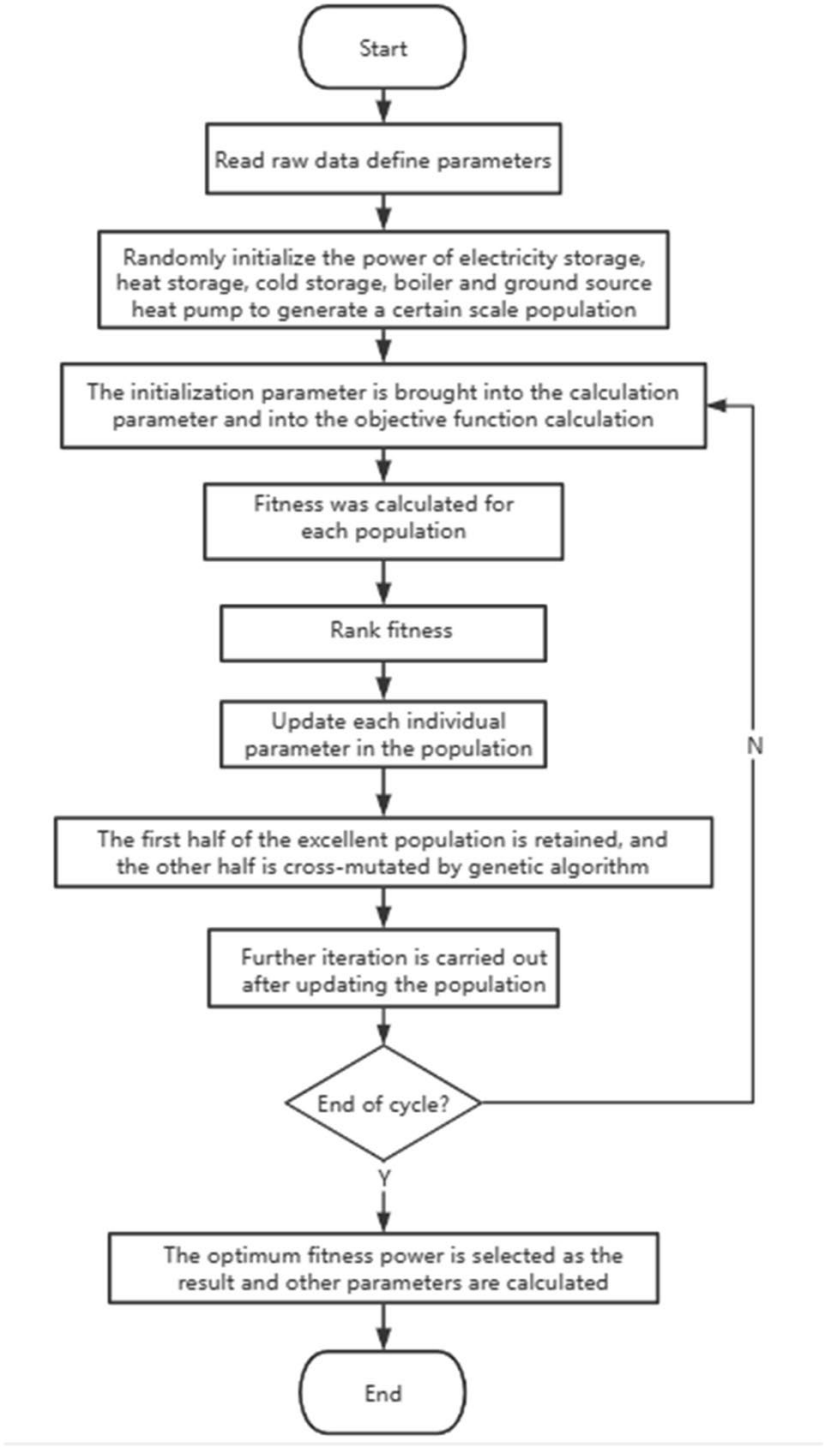
Flow chart of building integrated energy optimization.

This study focuses on the investigation of building integrated energy systems characterized by stable electricity demand and heating (cooling) requirements at the load side. The primary objective is to optimize and reconfigure the conventional supply structure, to attain the minimization of economic costs and carbon emissions while ensuring the fulfillment of cooling, heating, and electricity demands within the building. Figure 1 illustrates the flow chart depicting the optimization process of building integrated energy systems.

### Objective function

Optimizing and dispatching building integrated energy involves addressing a multi-objective quandary. In addition to considering system operating costs, environmental factors must also be taken into account. The attainment of both minimum system cost and carbon emission reduction is adopted as the objective function, wherein operation and maintenance costs encompass equipment, operational, and maintenance expenses, while carbon emission predominantly refers to carbon dioxide emissions.

$$\min F = \left\{ {f_{toc} ,f_{toe} } \right\}$$

In the context of this context,${\text{f}}_{{{\text{toc}}}} ,{\text{ and f}}_{{{\text{toe}}}}$ denote the variables representing operating and maintenance costs, as well as carbon emissions, respectively.

$$f_{B} = \mathop \sum \limits_{i = 1} L\left( i \right) + \mathop \sum \limits_{i = 1} M\left( i \right) + \mathop \sum \limits_{i = 1} N\left( i \right)$$

where,$f_{B}$ represents the cost of equipment,${\text{ L}}$ represents the electric vehicle; ${\text{M}}$ stands for gas turbine; ${\text{ N }}$ stands for ground source heat pump.

$$f_{G} = \mathop \sum \limits_{i = 1} \mathop \sum \limits_{{t \in R^{ + } }} M_{i} \times P_{M} \left( t \right) \times f_{g} \left( t \right)$$

where, $f_{G}$ represents the expenditure for the acquisition of gas. ${\text{M}}_{{\text{i}}}$ symbolizes the i gas turbine. ${\text{P}}_{{\text{M}}} \left( {\text{t}} \right)$ denotes the generated power output by the gas turbine in a specific period, t. ${\text{f}}_{{\text{g}}} \left( {\text{t}} \right)$ represents the cost associated with the procurement of natural gas during the mentioned period, t.

$$f_{Eb} = \mathop \sum \limits_{i = 1} \mathop \sum \limits_{{t \in R^{ + } }} N_{i} \times P_{N} \left( t \right) \times f_{{e_{1} }} \left( t \right) + \mathop \sum \limits_{i = 1} \mathop \sum \limits_{{t \in R^{ + } }} P_{i} \left( t \right) \times f_{{e_{2} }} \left( t \right)$$

where,${\text{ N}}_{{\text{i}}}$ symbolizes the I platform source heat pump, whereas ${\text{P}}_{{\text{N}}} \left( {\text{t}} \right)$ represents the power generated by the ground source heat pump in the specific period denoted as t. ${\text{f}}_{{{\text{e}}_{1} }} \left( {\text{t}} \right)$ signifies the price of electricity procured from the grid during the same period t. ${\text{P}}_{{\text{i}}} \left( {\text{t}} \right)$ represents the power consumption of unit i, while ${\text{f}}_{{{\text{e}}_{2} }} \left( {\text{t}} \right)$ denotes the price at which electricity is purchased from the grid during the aforementioned period.

$$f_{Es} = \mathop \sum \limits_{t = 1}^{T} P_{s} \left( t \right) \times f_{es} \left( t \right)$$

where ${\text{ P}}_{{\text{s}}} \left( {\text{t}} \right)$ and ${\text{f}}_{{{\text{es}}}} \left( {\text{t}} \right)$ represent the selling power and selling price of the t period respectively.

$$\begin{aligned} f_{MC} & = \mathop \sum \limits_{{i = 1,t \in R^{ + } }} \eta_{1} \times f_{E} \left( i \right) \times \left[ {P_{Ein} \left( t \right) - P_{Eout} \left( t \right)} \right] + \mathop \sum \limits_{{i = 1,t \in R^{ + } }} \eta_{2} \times f_{H} \left( i \right) \times \left[ {P_{Hin} \left( t \right) - P_{Hout} \left( t \right)} \right] \\ & \;\;\; + \mathop \sum \limits_{{i = 1,t \in R^{ + } }} \eta_{3} \times f_{C} \left( i \right) \times \left[ {P_{Cin} \left( t \right) - P_{Cout} \left( t \right)} \right] + \mathop \sum \limits_{i = 1} f_{p} \left( i \right) + \mathop \sum \limits_{i = 1} f_{w} \left( i \right) \\ \end{aligned}$$

where,${\upeta }_{1} ,{\upeta }_{2} ,{\upeta }_{3}$ represents the utilization frequency of electric vehicles, heat storage tanks, and cold storage tanks, ${\text{P}}_{{{\text{Ein}}}} \left( {\text{t}} \right),{\text{ P}}_{{{\text{Eout}}}} \left( {\text{t}} \right)$ represents the power stored and discharged by the electric vehicle during period “t”. ${\text{P}}_{{{\text{Hin}}}} \left( {\text{t}} \right),{\text{ P}}_{{{\text{Hout}}}} \left( {\text{t}} \right)$ denotes the heat storage and heat release power associated with the heat storage tank during the period “t”, and ${\text{P}}_{{{\text{Cin}}}} \left( {\text{t}} \right),{\text{ P}}_{{{\text{Cout}}}} \left( {\text{t}} \right)$ represents the power employed for storage and refrigeration in the storage tank during the period “t”. Finally, ${\text{f}}_{{\text{p}}} \left( {\text{i}} \right),{\text{ and f}}_{{\text{w}}} \left( {\text{i}} \right)$ signifies the fixed maintenance costs for photovoltaic and wind modules, respectively.

$$f_{{CO_{2} }} = \mathop \sum \limits_{{t \in R^{ + } }} E_{t,t + 1}^{g} \times \eta_{{g,co_{2} }} + \mathop \sum \limits_{{t \in R^{ + } }} E_{t,t + 1}^{e} \times \eta_{{e,co_{2} }}$$

where,${\text{ E}}_{{{\text{t}},{\text{t}} + 1}}^{{\text{g}}} ,{\text{ E}}_{{{\text{t}},{\text{t}} + 1}}^{{\text{e}}}$ respectively denote the rate of natural gas and electricity consumption per unit of time. ${\upeta }_{{{\text{g}},{\text{co}}_{2} }} ,{\upeta }_{{{\text{e}},{\text{ and co}}_{2} }}$ respectively signify the conversion factors for carbon dioxide emissions resulting from the combustion of natural gas and the generation of thermal power from electricity.

### Constraints

#### Constraints on the balance of cooling, heating, and electricity

The equilibrium between energy supply and demand within the system primarily encompasses the balance of electrical power, thermal power, and cooling power.

$$\begin{aligned} P_{b} (t) - P_{s} (t) + P_{M} (t) + P_{PV} (t) + P_{WT} (t) + P_{Eout} (t) & = P_{N} (t) + P_{Ei} (t) + P_{Ein} (t) \\ \eta_{h1} \times P_{M} (t) + \eta_{h2} \times P_{N} (t) + P_{Hout} (t) & = P_{Hi} (t) + P_{Hin} (t) \\ \eta_{c} \times P_{N} \left( t \right) + P_{Cout} \left( t \right) & = P_{Ci} \left( t \right) + P_{Cin} \left( t \right) \\ \end{aligned}$$

where,${\text{ P}}_{{\text{b}}} \left( {\text{t}} \right),{\text{ P}}_{{{\text{PV}}}} \left( {\text{t}} \right),{\text{ P}}_{{{\text{WT}}}} \left( {\text{t}} \right)$ signifies the purchased power, photovoltaic power, and wind power during the period t, each respectively. ${\text{P}}_{{{\text{Ei}}}} \left( {\text{t}} \right),{\text{ P}}_{{{\text{Hi}}}} \left( {\text{t}} \right),_{{}} {\text{ and PCI }}\left( {\text{t}} \right)$ respectively denote the periods associated with the user's electricity, heat, and cooling load. $\eta_{h1}$ represents the heating efficiency of the waste heat boiler in the gas boiler system, $\eta_{h2} ,\eta_{c}$ and represents the heating and cooling efficiency of the ground source heat pump.

#### Constraints on the energy storage system

The energy storage system mainly includes electric storage, heat storage, and cold storage devices. The energy storage device does not generate and consume energy spontaneously but only realizes energy transfer on a time scale. Take the electric storage device as an example:

$$S_{E}^{min} \le S_{E} \left( t \right) \le S_{E}^{max}$$

$$0 \le P_{Ein} \left( t \right) \le \lambda_{e} P_{in}^{max}$$

$$0 \le P_{Eout} \left( t \right) \le \left( {1 - \lambda_{e} } \right)P_{out}^{max}$$

where,${\text{ S}}_{{\text{E}}}^{{{\text{min}}}} { }$ and ${\text{S}}_{{\text{E}}}^{{{\text{max}}}}$ are employed to signify the lower and upper bounds of the electric storage device, respectively. Correspondingly, ${\text{P}}_{{{\text{in}}}}^{{{\text{max}}}}$ and ${\text{P}}_{{{\text{out}}}}^{{{\text{max}}}}$ are utilized to denote the maximum charging power and maximum discharge power, respectively. $\lambda_{{\text{e}}}$ is indicative of the charge and discharge status of the storage device, constrained between the range of [0, 1].

#### Constraints on device running

In scenarios where the gas turbine's output is relatively low, the economic cost is considered, and a shutdown threshold is established to prevent inefficiencies arising from low generation efficacy. Accordingly, the gas turbine's output must surpass the predetermined shutdown power. The constraints are outlined as follows

$$\phi_{M} \times P_{M}^{max} \le P_{M} \left( t \right) \le P_{M}^{max}$$

where, $\phi_{{\text{M}}}$ represents the shutdown factor associated with a gas turbine, while ${\text{P}}_{{\text{M}}}^{{{\text{max}}}}$ denotes the maximum output power generated by the gas turbine. On the other hand, other devices do not impose any shutdown constraints. To illustrate, let us consider the ground source heat pump, which is subject to the following operational constraints:

$$0 \le P_{N} \left( t \right) \le P_{N}^{max}$$

where ${\text{ P}}_{{\text{N}}}^{{{\text{max}}}}$ symbolizes the maximal power output of the ground source heat pump.

#### Constraints on power purchase and sale

$$\begin{gathered} P_{b}^{\min } \le P_{b} (t) \le P_{b}^{\max } \hfill \\ P_{s}^{\min } \le P_{s} (t) \le P_{s}^{\max } \hfill \\ P_{b} (t)P_{s} (t) = 0 \hfill \\ \end{gathered}$$

where,${\text{ P}}_{{\text{b}}}^{{{\text{min}}}}$ and ${\text{P}}_{{\text{b}}}^{{{\text{max}}}}$ are used to signify the minimum and maximum power purchased, respectively. Similarly, ${\text{P}}_{{\text{s}}}^{{{\text{min}}}}$ and ${\text{P}}_{{\text{s}}}^{{{\text{max}}}}$ are employed to denote the minimum and maximum power sold, respectively.

## Building integrated energy optimization scheduling system algorithm

### Multi-objective whale optimization algorithm

**Figure 2:**
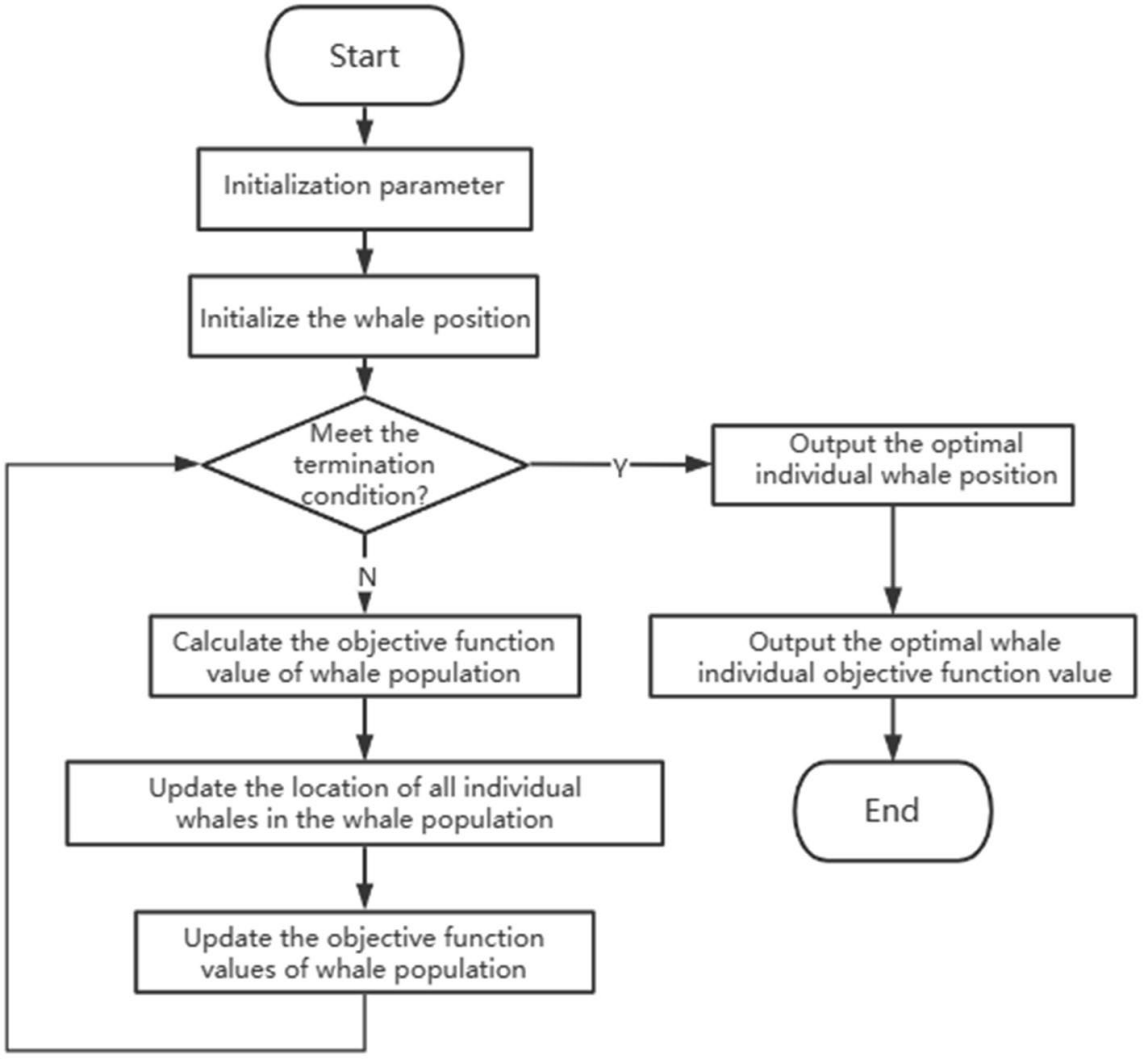
Flowchart of whale algorithm.

The optimization scheme suggested in this research for building integrated energy scheduling necessitates the simultaneous consideration of economic and environmental aspects, thereby establishing it as a multi-objective optimization problem. The utilization of the Whale Optimization Algorithm (WOA) stems from the imitation of humpback whale groups' hunting behavior in nature. Through a series of sequential actions including searching, encircling, hunting, and attacking prey, the WOA aims to accomplish the objective of optimization search. The conventional WOA comprises distinct stages such as prey encircling, spiral bubble formation, and prey detection. A visual representation of the WOA principle is illustrated in Fig. 2.

### Based on improved multi-objective whale optimization algorithm

To enhance the convergence speed and prevent the Whale Algorithm from converging to local optima, this study incorporates the Genetic Algorithm (GA) and refines its fitness model. This integration serves to augment the global search capability and convergence speed of the Whale Algorithm, while concurrently ensuring the population's diversity and elevating the algorithm’s accuracy.

#### Whale algorithm

**Figure 3:**
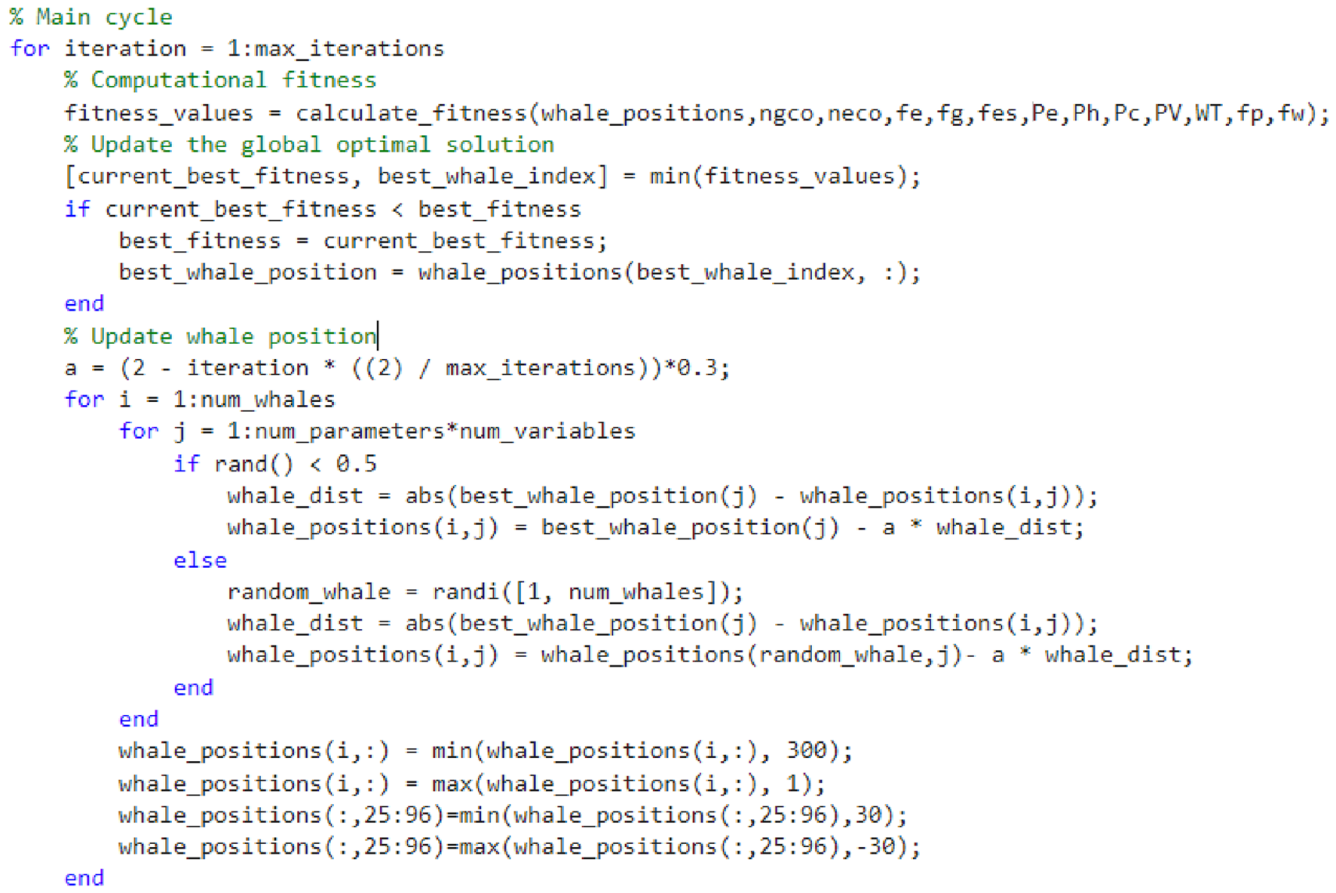
Initial and updated optimal solutions of whale algorithm.

After setting parameters, the whale position and fitness are first initialized, and when the global optimal solution and the whale optimal position are updated later, it is easy to fall into the local optimal. Therefore, optimization methods such as genetic algorithm are introduced later. Figure 3 shows the process of the whale algorithm initializing and updating the optimal solution.

#### Cross operation

**Figure 4:**
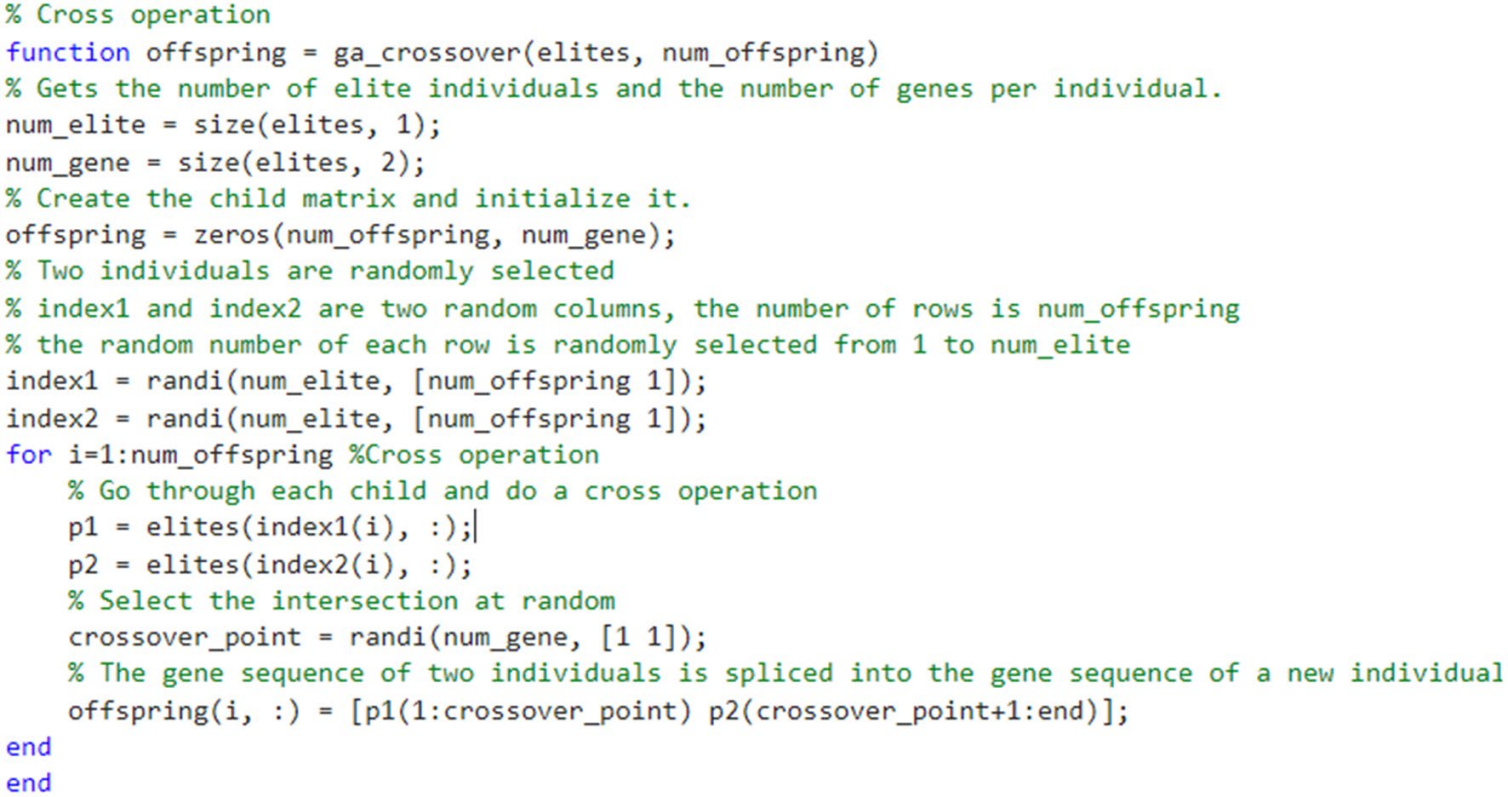
Genetic algorithm to optimize cross operation.

Crossover is the driving force of genetic algorithm, resulting in the structured and accidental exchange of genetic material between solutions, and it is possible that special chromosomes produce better chromosomes, and random crossover is a necessary process of genetic algorithm. Figure 4 shows the random crossover of genetic algorithm.

#### Mutation operation

**Figure 5:**
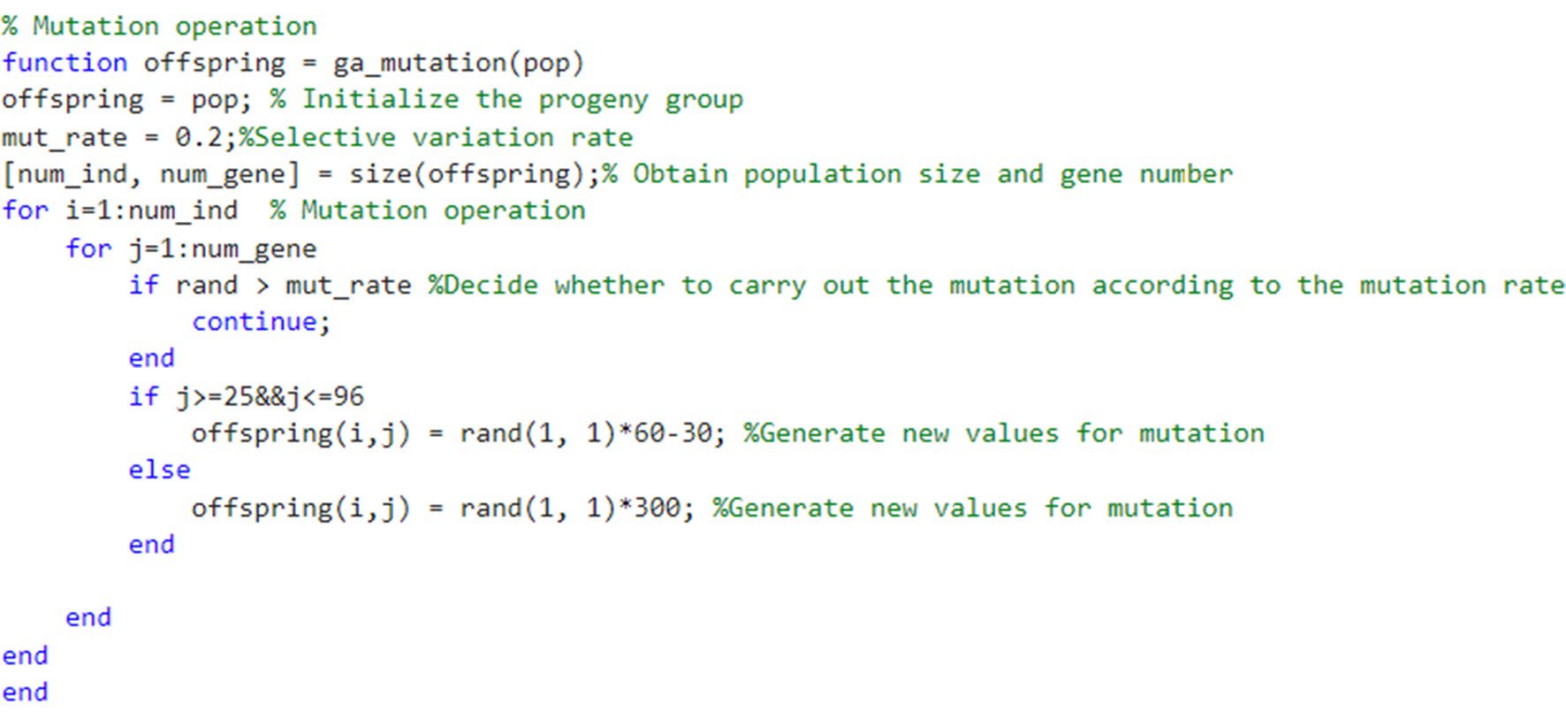
Genetic algorithm to optimize mutation operation.

After cross operation, the chromosome is maintained in the mutation process. This operation is designed to prevent the genetic algorithm from falling into local minima. Mutations have two main purposes, one is to restore extinct genetic material, and the other is to destroy genetic information. Appropriate mutation probabilities can maintain the diversity of the population^23^. Figure 5 shows the compilation operation process.

#### Genetic algorithm fitness function improvement

To address the necessity of balancing the global and local search capabilities within the Whale Algorithm, this study proposes the integration of an adaptive weight mechanism alongside the calculation of an individual’s relative position within the entire population to enable adaptive position updates. The conventional adaptive weight approach solely divides the whale individuals into best, worst, and average groups, wherein the classification of most general group individuals remains inadequate. In this paper, an enhanced adaptive function fitness model is introduced, enabling a more detailed classification of general group individuals into superior, inferior, and ordinary groups. The specific workflow proceeds as follows: the fitness of individual whales in the population is ranked in ascending order, generating the respective averages for the preceding, succeeding, and complete subsets. Subsequently, the individual whales are classified based on their current fitness and allocated corresponding weights.

1. In circumstances where the fitness value f(i) of an individual whale falls below a certain threshold, it signifies that said individual belongs to the optimal group within the population. In response, a minor weight value is assigned to reinforce its local search proficiency.
2. When the fitness value f(i) of an individual whale falls within the interval [lower bound] and [upper bound], it signifies that said individual belongs to the general group within the population. Consequently, a weight value of 1 is assigned, ensuring that the individual can continue approaching the optimal position through the algorithm mechanism.
3. When comparing the fitness value f(i) of an individual whale with that of the optimal whale, a situation arises wherein the former surpasses the latter, signifying the individual's classification as belonging to the inferior group within the population. In this case, it becomes imperative to reinforce its capability for global exploration. This is achieved by selecting a larger or smaller weight, with a probability of 50%, to facilitate the prompt evasion of local optima.

To achieve a more refined classification of general group individuals in (2), the individuals are subjected to a detailed categorization, enabling a clearer differentiation between superior and inferior individuals within this group. This improved classification is facilitated by the introduction of an adaptive function.

$$Fit\left( i \right) = \alpha \times \gamma_{R} \left( D \right) + \beta \times \frac{\left| C \right| - \left| R \right|}{{\left| C \right|}}$$

where, refers to the coefficient which takes values within the range of [0,1]. denotes the length of the feature subset, while C represents the total number of feature attributes. Additionally, signifies the degree of dependence of the conditional attribute on the decision attribute.

$$d_{i,j} = \left| {Fit_{i} ,Fit_{j} } \right|$$

Calculate the difference between individual whale I and the best and worst individual in the group ${\text{d}}_{{{\text{i}},{\text{good}}}}$ and ${\text{d}}_{{{\text{j}},{\text{inf}}}}$. If yes ${\text{d}}_{{{\text{i}},{\text{ good}}}} > \exp \left( { - \frac{{{\text{Iter}}}}{{{\text{Iter}}_{{{\text{max}}}} }}{\text{d}}_{{{\text{j}},{\text{inf}}}} } \right)$, it is the better group. If yes ${\text{d}}_{{{\text{i}},{\text{ good}}}} \le \exp \left( { - \frac{{{\text{Iter}}}}{{{\text{Iter}}_{{{\text{max}}}} }}{\text{d}}_{{{\text{j}},{\text{inf}}}} } \right)$, it is the worse group.

$$\omega (i) = \left\{ \begin{gathered} \omega_{1} + (\omega_{2} - \omega_{1} ) \times \frac{{f(i) - f_{\min } }}{{\mu a_{1} - f_{\min } }},f(i) \le \mu a_{1} \hfill \\ \omega_{1} - (\omega_{2} - \omega_{1} ) \times \frac{{f(i) - f_{\max } }}{{\mu a_{3} - f_{\max } }},f(i) \ge \mu a_{3} ,k \ge 0.5 \hfill \\ \omega_{2} + (\omega_{3} - \omega_{2} ) \times \frac{{f(i) - f_{\max } }}{{\mu a_{3} - f_{\max } }},f(i) \ge \mu a_{3} ,k \le 0.5 \hfill \\ \omega_{2} + (\omega_{2} - \omega_{1} ) \times \frac{{f(i) - f_{\max } }}{{\mu a_{2} - f_{\max } }},\mu a_{1} \le f(i) \le \mu a_{2} ,k \ge 0.5 \hfill \\ \omega_{2} + (\omega_{2} - \omega_{1} ) \times \frac{{f(i) - f_{\min } }}{{\mu a_{1} - f_{\min } }},\mu a_{1} \le f(i) \le \mu a_{2} ,k \le 0.5 \hfill \\ \omega_{3} + (\omega_{3} - \omega_{2} ) \times \frac{{f(i) - f_{\max } }}{{\mu a_{3} - f_{\max } }},\mu a_{2} \le f(i) \le \mu a_{3} ,k \ge 0.5 \hfill \\ \omega_{3} + (\omega_{3} - \omega_{2} ) \times \frac{{f(i) - f_{\min } }}{{\mu a_{2} - f_{\min } }},\mu a_{2} \le f(i) \le \mu a_{3} ,k \le 0.5 \hfill \\ \end{gathered} \right.$$

where ${\text{f}}\left( {\text{i}} \right)$ represents the fitness value of the first whale. Moreover, ${\text{k}}$ is a random number that falls within the inclusive range of [0,1]. Additionally, $\omega_{1} ,\;\omega_{2} ,\;\omega_{3}$ denotes the adaptive weight limit, while $\omega_{3} > \omega_{2} > \omega_{1}$ refers to a variable. Utilizing the aforementioned adaptive weights, the position update formula of the whale algorithm is improved to:

$$X\left( {t + 1} \right) = \left\{ {\begin{array}{*{20}l} {\omega \times X^{*} \left( t \right) - A \times D,\;\;p > 0.5} \hfill \\ {\omega \times X^{*} \left( t \right) + D^{\prime} \times e^{bl} \cos (2\pi l),\;p \le 0.5} \hfill \\ \end{array} } \right.$$

where, is a stochastic variable, uniformly distributed in the interval [0,1].

**Figure 6:**
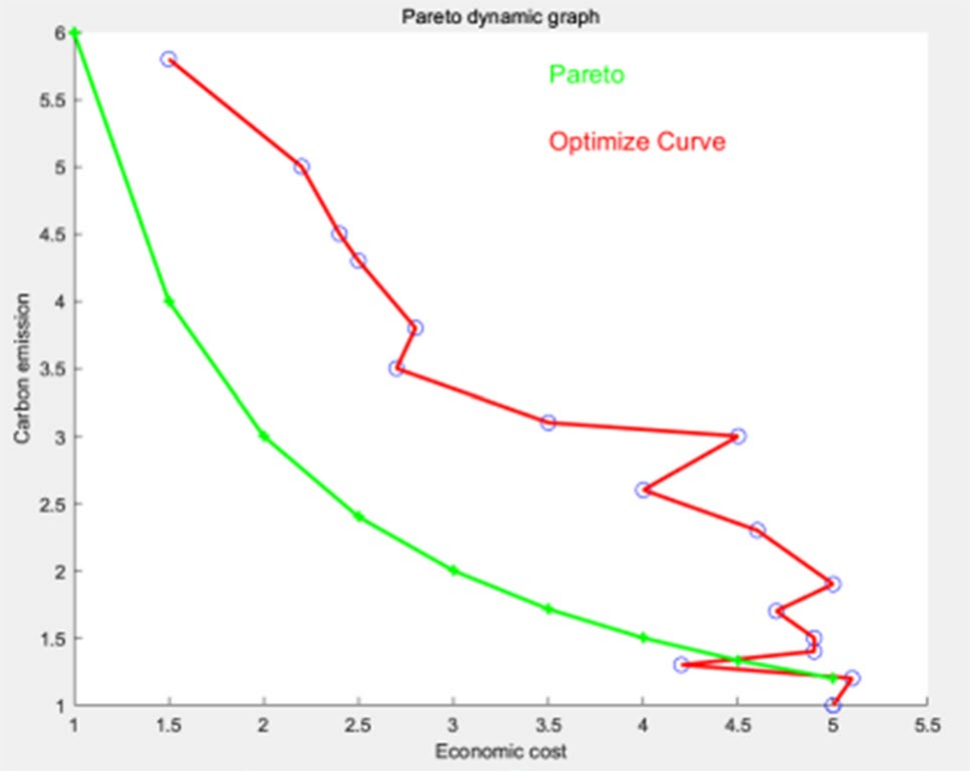
No algorithm used.

**Figure 7:**
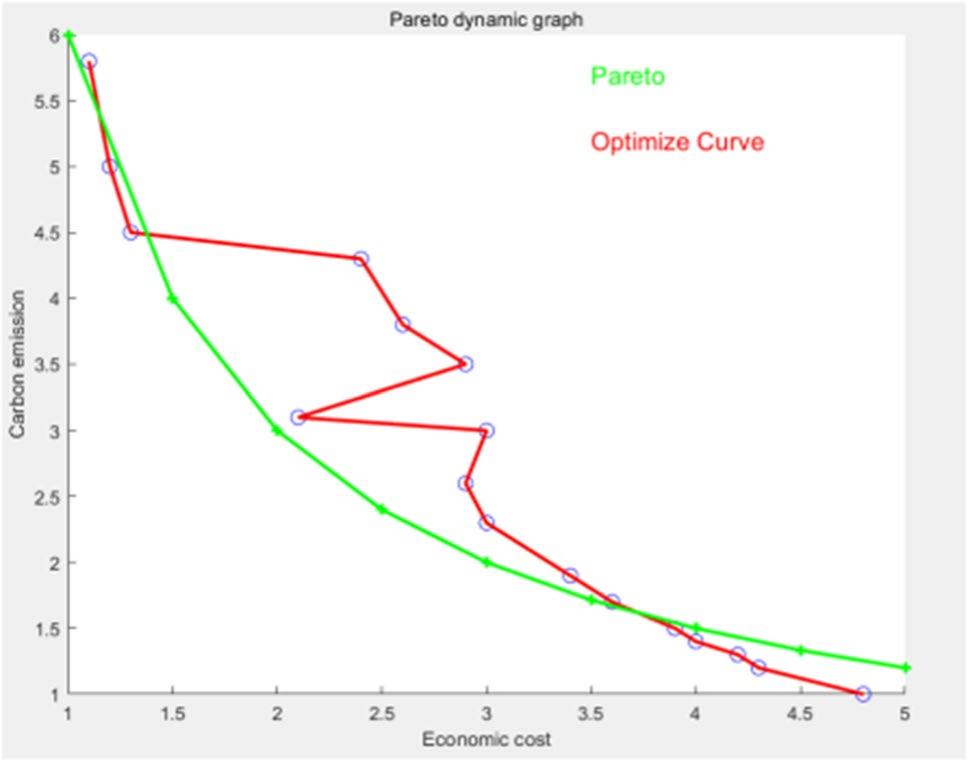
Algorithm used for 100 iterations.

**Figure 8:**
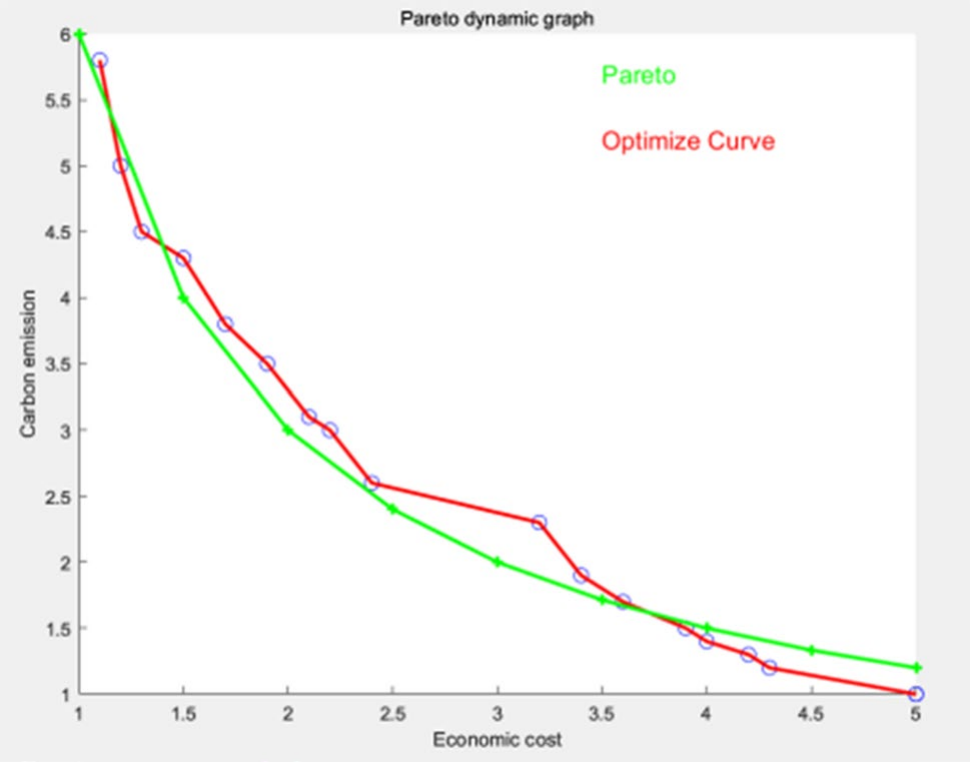
Algorithm used for 500 iterations.

The results depicted in Figs. 6, 7, and 8 demonstrate the efficacy of the algorithm's enhancements. When considering an objective function with two objectives and a defined opposition, the curve of the optimal solution set closely approximates the Pareto frontier. It is evident that the algorithm successfully achieves a close approximation to the Pareto frontier, thereby significantly enhancing its accuracy.

## Result

**Figure 9:**
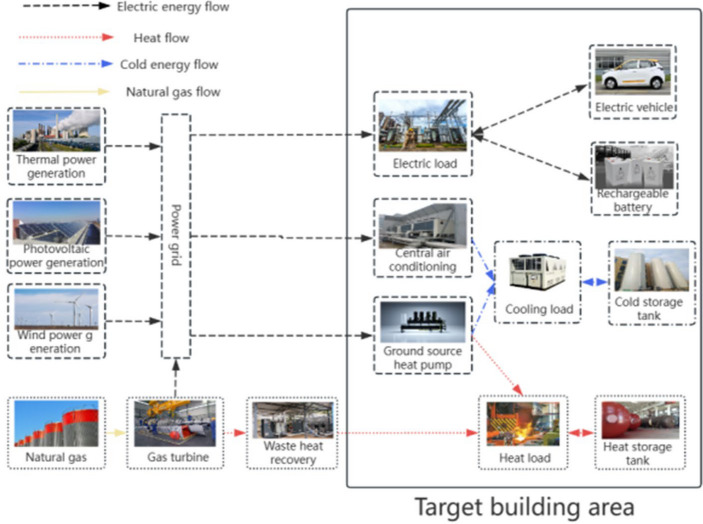
Integrated energy system structure diagram.

In order to evaluate the effectiveness of the above model and algorithm and comprehensively solve the problems related to economic cost and environmental pollution, the example adopts the improved multi-objective genetic whale algorithm. The parameter setting and operator selection are consistent with the above description, and the convergence speed and accuracy as well as the reliability of the results are demonstrated by comparing with other algorithms. This study takes the regional complex building as an example to optimize the simulation. Specifically, we chose a single day as the focus of our analysis. The building primarily relies on grid power, gas turbines, and new energy generation for its electricity supply. As for heat energy, the waste heat boiler, central air conditioning, and ground source heat pump play prominent roles by utilizing micro-gas turbines to absorb heat from high-temperature flue gas. Additionally, the cold energy is sourced from the central air conditioning and ground source heat pump. The system configuration is depicted in Fig. 9.

### Basic data

In this paper, load data of a comprehensive commercial building in a province of China is collected, initial data is cleaned, abnormal data is deleted, and blank data is supplemented by K-nearest neighbor Imputation. Purchase and sale prices, heating and cooling prices and incentive subsidy prices ref. 18, while Table 1 provides an overview of the operational parameters of the equipment.

**Table 1:** Equipment operating parameters.

Device name	Argument
Rated power of gas turbine	100 kw
Rated power of waste heat boiler	100 kw
Rated power of ground source heat pump	40 kw
Rated power of central air conditioning	50 kw
Rated power of electric vehicles	50 kw
Battery capacity	120 kw
Capacity of heat storage tank	200kw
Capacity of cold storage tank	200kw
Gas turbine power generation efficiency	0.55
Charging and discharging efficiency of heat storage tank	0.95
Charging and discharging efficiency of cold storage tank	0.95
Battery charging and discharging efficiency	0.95

### Algorithm comparison

The main experimental tools for building energy optimization scheduling are matlab, custom programming algorithms, and general optimization packages.

In order to verify the feasibility of the proposed algorithm in building comprehensive energy optimization scheduling, algorithms were compared for the same scenario.

Algorithm 1: The improved genetic whale algorithm proposed in this paper is used for energy scheduling, and the adjustment of gas turbines, wind power generation and energy storage equipment is realized by analyzing demand data and response data.

Algorithm 2: The improved whale algorithm is used for energy scheduling, and its data analysis is consistent with algorithm 1.

**Figure 10:**
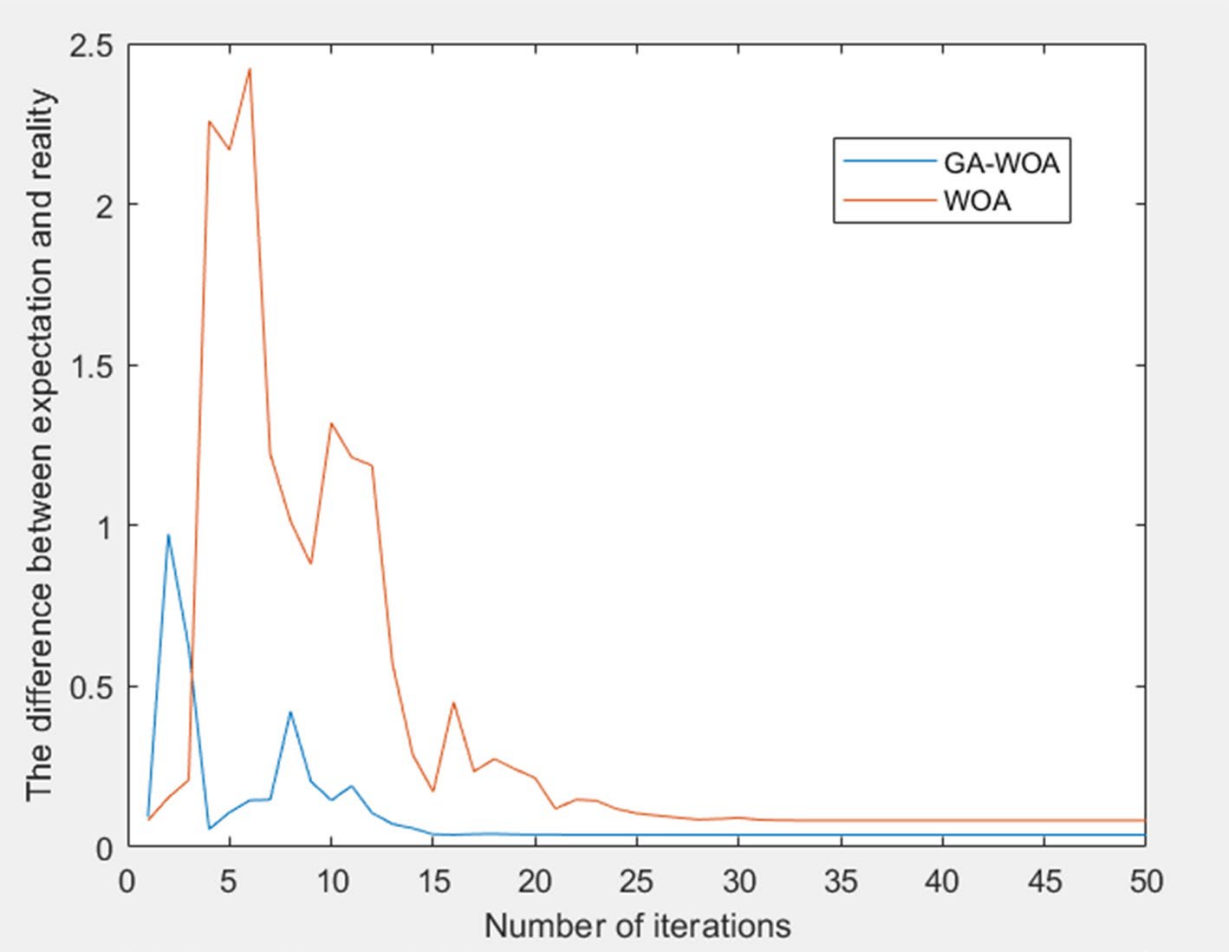
Compares the iteration speed and accuracy of the algorithm.

Figure 10 presents a comparative analysis of the iteration speed and accuracy between the original Whale Algorithm and the enhanced Genetic Whale Algorithm. The simulation results indicate that the improved algorithm not only enhances the iteration speed but also exhibits a substantial improvement in accuracy.

Table 2 shows the comparison of economic costs and carbon emissions of power grid procurement scheduling and integrated energy system scheduling under different algorithms in wind power and photovoltaic forecast scenarios.

**Table 2:** Scheduling comparison under different algorithms.

Algorithm	Power purchasing and dispatching cost/yuan	Comprehensive energy dispatch cost/yuan	Economic cost/yuan	Carbon emissions/ton
Algorithm1	2201.012	820.545	3021.557	1.790
Algorithm2	2312.177	802.276	3114.453	1.881

### Result analysis

**Figure 11:**
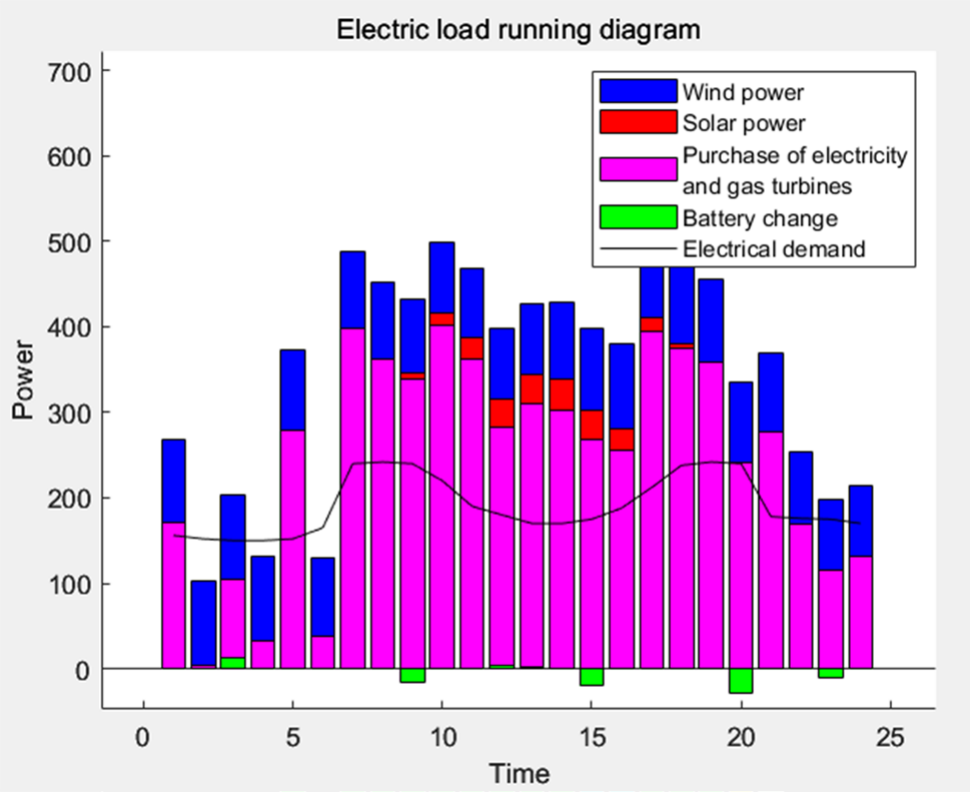
Power load running balance diagram.

**Figure 12:**
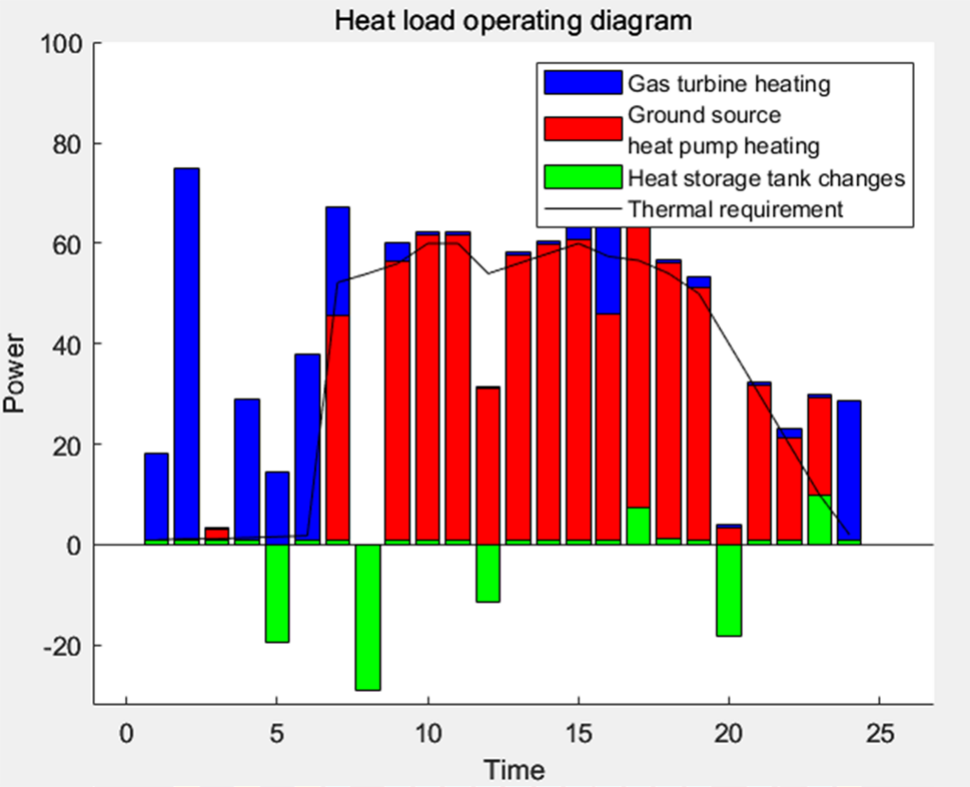
Heat load operation diagram.

**Figure 13:**
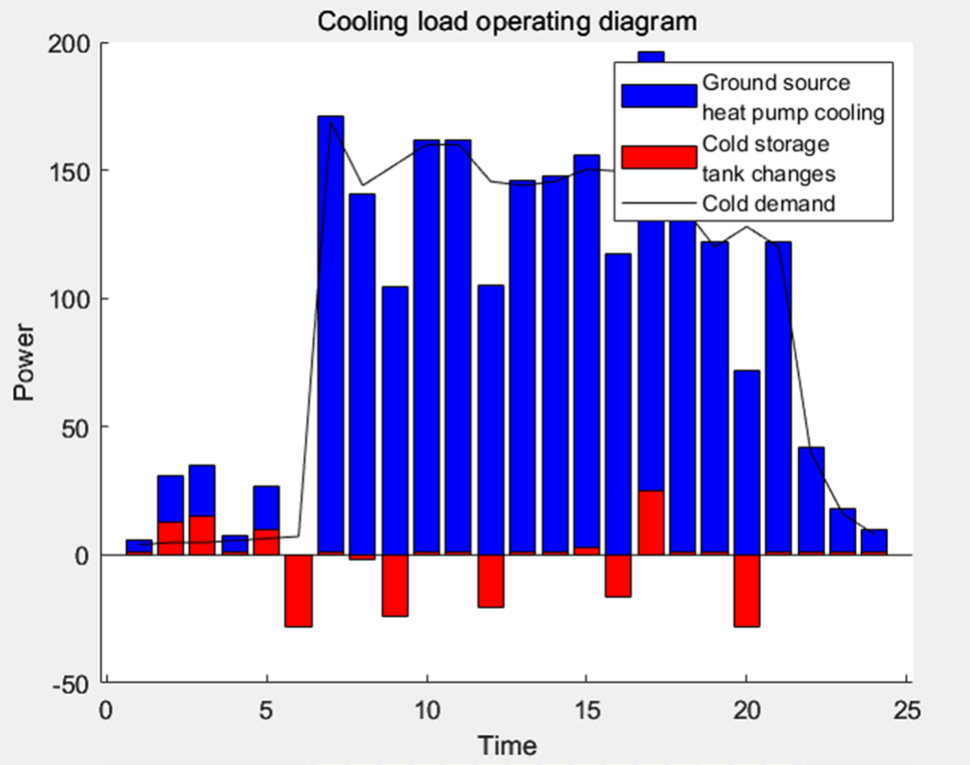
Cooling load running balance diagram.

**Figure 14:**
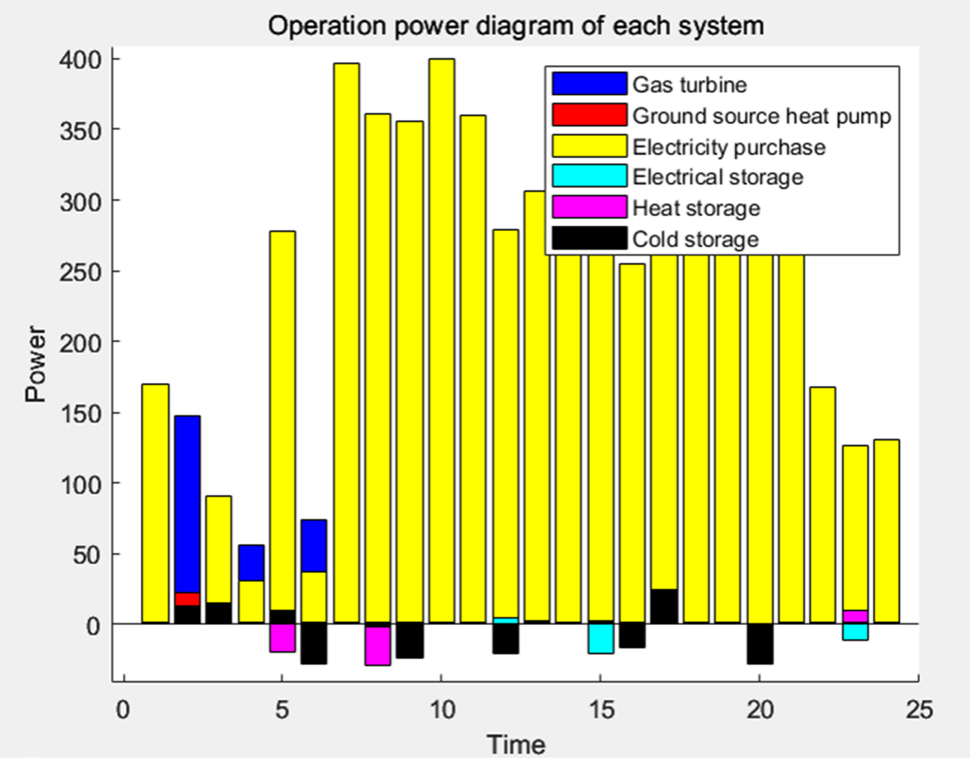
Operation power diagram of each system.

Following a thorough analysis of the scheduling outcomes, the resulting electrical load, thermal load, cooling load, and power diagram are visually represented in Figs. 11, 12, 13, and 14, respectively.

Based on the representation in Fig. 9, it can be observed that the main sources of power demand for the building are the main supply and gas turbine generation. This can be attributed to the stable supply from the mains grid, accompanied by the advantages of high power quality and low maintenance costs associated with the full operation of micro-gas turbines. Moreover, photovoltaic power generation is primarily concentrated between 9 and 16 h, with minimal involvement in power supply during other periods. On the other hand, wind power generation remains relatively stable throughout the day. The building relies on the battery for electricity provision in scenarios where the power supply is insufficient, while surplus power is stored during periods of excess supply.

The analysis of Fig. 10 reveals that the heat load primarily relies on waste heat absorption and the ground source heat pump. Throughout the daytime, the ground source heat pump predominantly provides the requisite heat supply, with a minor contribution from the boiler. However, during nighttime, the majority of the heat requirement is fulfilled by the boiler. Additionally, a surplus of heat energy is observed between 12 and 14 noon, which is stored in the heat storage tank for subsequent heating purposes at other times.

According to the representation in Fig. 11, the primary origins contributing to the cooling load comprise the ground source heat pump and central air conditioning systems. From 8 to 21 h, a robust cooling demand persists through the day. To fulfill this demand, intermittent cooling supply is employed, alongside periodic replenishment of the cooling load within the cold storage tank, which ensures the fulfillment of the cooling load requirements during the night-time period.

As depicted in Fig. 12, the primary source of electricity supply is derived from the mains, resulting in a higher demand for purchased power during most daylight hours. However, during peak solar irradiation at noon, photovoltaic power generation yields a surplus, leading to a reduction in purchased power and enabling the heat storage tank to accumulate a substantial portion of the generated heat. During nighttime, the electrical load demand significantly decreases, resulting in excess power generation across all systems. In addition to storing a portion of this excess load, the remaining surplus power is exported back to the grid.

## Conclusion

By utilizing an enhanced multi-objective whale algorithm, this study formulates a model for optimizing the scheduling of building-integrated energy systems. Through the incorporation of simulation cases, the paper derives the subsequent conclusions: this study addresses the conflicting objectives of minimizing economic costs and carbon emissions through the formulation of an objective function that simultaneously satisfies both criteria. A Pareto dynamic optimization approach is employed to obtain the curve representing the optimal solution set. Moreover, an enhanced multi-objective genetic whale algorithm is proposed in this research. Simulation results show that the improved algorithm effectively reduces the sensitivity of whale algorithm to local optimization, and improves the global search ability and convergence speed. In the case of using the algorithm, the daily cost is saved 92.896 yuan, and the carbon emission is reduced by 0.091 tons. In addition, the enhanced algorithm has strong adaptability in solving multi-objective scheduling problems.

The future work should further test the application and schedule the acquisition analysis for a variety of buildings to improve the universality of the algorithm. In addition, it can be applied by combining various optimization methods to continuously improve the speed and accuracy of the algorithm in solving practical problems.

## Data Availability

All data generated or analyzed during this study are included in this published article.
